# Effects of extrusion of rice bran on performance and phosphorous bioavailability in broiler chickens

**DOI:** 10.1186/s40781-015-0059-z

**Published:** 2015-07-20

**Authors:** Ali Akbar Zare-Sheibani, Masoud Arab, Mohammad Javad Zamiri, Mohammad Reza Rezvani, Mohammad Dadpasand, Farhad Ahmadi

**Affiliations:** Present Address: Golpooneh Safahan Co., Isfahan Science & Technology Town, Sheikh Bahai Building, Isfahan, Iran; Department of Animal Science, College of Agriculture, Shiraz University, 71441-65186 Shiraz, Iran

**Keywords:** Rice bran, Extrusion, P availability, Performance, Broiler chicken

## Abstract

**Background:**

Rice bran is a by-product of the rice-milling process, which remains largely underutilized; however, efficient processing treatments may improve its feeding value for chickens. This is of great economic and environmental importance, as this can lower the production costs, and offer an opportunity for valorization of a low-quality agricultural by-product, to a high-value feed source.

**Methods:**

This experiment was conducted to study the effect of extruded rice bran on performance and phosphorous (P) bioavailability in broiler chickens. In a completely randomized design, 200 seven-day-old broilers (Cobb 500) were allotted to five treatments with five replicates per treatment and 8 chicks per replicate, and fed with their respective diet during the starter (8 to 21 days) and grower (22 to 42 days) periods. Diets were a basal corn-soybean based diet (T1), or diets containing 20 % rice bran (T2), 30 % rice bran (T3), 20 % extruded rice bran (T4), or 30 % extruded rice bran (T5).

**Results:**

Birds feeding on T4 and T5 diets had a higher body weight gain and lower feed-to-gain ratio compared to those feeding on T2 and T3 diets (*p* < 0.05). Birds receiving diets containing extruded rice bran had higher total P availability and tibia ash content, as compared with those receiving diets containing un-extruded rice bran (*p* < 0.05). Relative weight of the pancreas was higher in birds receiving T2 and T3 diets.

**Conclusions:**

The results confirmed the beneficial effect of extrusion treatment of rice bran on performance and P availability in broilers. Up to 30 % extruded rice bran may be included in the broiler diet without apparent adverse effects on the performance.

## Background

Rice bran, an important by-product of the rice milling industry, has the potential to be used as an alternative to grains in the poultry diet [1]. It contains 15 − 22 % oil, 11 − 17 % protein, 6 − 14 % fiber, 10 − 15 % moisture, and 8 − 17 % ash [2]; however, the presence of anti-nutritional factors such as phytic acid [3], trypsin inhibitor and hemagglutinin [4], high fiber content [5], and instability during storage [6] limit its use in poultry nutrition. Pancreatic hypertrophy [7, 8], depressed intestinal amylase activity [8], and reduction in feed consumption [9] and growth [10] have been reported as the side effects of feeding raw rice bran to broilers.

Approximately 90 % of P in rice bran is in the form of phytic acid and phytate chelated with other elements; phytate not only does reduce P availability, but also decreases the absorption of elements such as zinc, iron, calcium and magnesium [11]. In addition, it decreases protein digestibility and energy use through inhibition of the enzymes such as pepsin, trypsin and α-amylase [12, 13]. Rice bran also contains lipase and peroxide which are released during rice polishing and cause lipid oxidation [2, 14]. Therefore, it is necessary to stabilize the rice bran by effective pretreatments to limit the undesirable reactions [2]. The extrusion cooking technology, a thermal/mechanical pretreatment applying heat treatment in the presence of moisture, is an effective processing method causing physico-chemical and nutritional modifications of the food constituents, such as permanently denaturing lipases [15–17]. Use of extruded rice bran in the diet of broilers, compared with raw and roasted rice bran, significantly increased fat digestibility which was attributed to lipase and trypsin inhibitor inactivation [1, 15]. In addition, use of 10, 20 and 30 % extruded rice bran in broiler diets, compared with raw and roasted rice bran, increased weight gain and feed consumption [6]. Sayre et al. [18] showed that feeding 600 g extruded rice bran/kg feed compared with raw rice bran, improved broiler weight gain and feed efficiency during the first two weeks of production, but after the second week, the performance of chicks fed the extruded rice bran was depressed.

The poultry responses to extrusion processing of rice bran are not conclusive, especially P availability, and more research is warranted to elucidate the dietary effect of extruded rice bran on performance and P availability in broiler chicks. Therefore, the present experiment was conducted to determine the effect of extruded rice bran on performance and P availability in broiler chicks.

## Methods

### Preparation of rice bran and extrusion process

Rice bran, provided within 12 h of milling, was extruded at 125 °C and 40-bar pressure by using a high-capacity single-helix extruder (Andritz Extruder, Model EX620, Denmark). The length and diameter of the transport screws were 1600.0 and 210.0 mm, respectively, having a length/diameter ratio of 7.62. The passage time was 10 s. The extruder barrel temperature and the screw speed were controlled by a computer connected to the extruder. Production capacity of the extruder was 2–9 TPH.

### Experimental birds and diets

The study protocol was approved by the Animal Ethics Committee of Shiraz University and the birds were managed in compliance with the Shiraz University animal care guidelines and policies. Two-hundred seven-day-old broilers (Cobb 500), allotted to five dietary treatments with five replicates per treatment and 8 chicks per replicate, and reared in floor pens (100 cm × 100 cm). Diets consisted of a control diet based on corn and soybean (T1), and diets containing 20 or 30 % rice bran (T2 and T3, respectively) and 20 or 30 % extruded rice bran (T4 and T5, respectively). The diets, formulated according to the recommendations of the National Research Council [19], were fed as starter (8 to 21 d of age) and grower (22 to 42 d of age) diets. Ingredients and nutrient composition of the diets are shown in Table 1. All diets were identical in terms of density of metabolizable energy (ME), crude protein, calcium, available P, lysine, methionine and methionine + cystine. In the first week, a balanced corn-soybean diet was fed. The chicks had ad libitum access to feed and water.

**Table 1:** Composition (g/kg, as fed basis) of the experimental diets^a^

Ingredients and composition	Starter					Finisher				
	T1	T2	T3	T4	T5	T1	T2	T3	T4	T5
Corn	592.1	407.6	315.2	407.6	315.2	648.4	463.8	371.5	463.8	371.5
Soybean meal, (480 g crude protein/kg)	357.3	334.5	323.0	334.5	323	300.1	277.1	265.7	277.1	265.7
Raw rice bran	0	200	300	0	0	0	200	300	0	0
Extruded rice bran	0	0	0	200	300	0	0	0	200	300
Soybean oil	13	21.3	25.5	21.3	25.5	17.8	26.2	30.3	26.2	30.3
Dicalcium phosphate	14.4	13.2	12.6	13.2	12.6	13.3	12.1	11.5	12.1	11.5
Calcium carbonate	12.5	13.1	13.5	13.1	13.5	11.7	12.4	12.7	12.4	12.7
Salt	4.2	3.9	3.8	3.9	3.8	3.1	2.9	2.8	2.9	2.8
Vitamin premix^b^	2.5	2.5	2.5	2.5	2.5	2.5	2.5	2.5	2.5	2.5
Mineral premix^c^	2.5	2.5	2.5	2.5	2.5	2.5	2.5	2.5	2.5	2.5
DL-methionine (990 g methionine/kg)	1.5	1.4	1.4	1.4	1.4	0.6	0.5	0.5	0.5	0.5
Calculated nutrients^d^										
Crude protein	208	208	208	208	208	188	188	188	188	188
ME, kcal/kg	2900	2900	2900	2900	2900	3000	3000	3000	3000	3000
Lys	11.2	11.2	11.2	11.2	11.2	9.8	9.8	9.9	9.8	9.9
Met	4.8	4.7	4.7	4.7	4.7	3.6	3.6	3.6	3.6	3.6
Met + Cys	8.2	8.2	8.2	8.2	8.2	6.8	6.8	6.8	6.8	6.8
Calcium	9.1	9.1	9.1	9.1	9.1	8.4	8.4	8.4	8.4	8.4
Total P	6.7	8.8	9.8	8.8	9.8	6.3	8.4	9.4	8.4	9.4
Available P	4.1	4.1	4.1	4.1	4.1	3.8	3.8	3.8	3.8	3.8
Sodium	1.8	1.8	1.8	1.8	1.8	1.4	1.4	1.4	1.4	1.4

^a^Starter (8–21 d); finisher (22–42 d)

T1 control, T2 20 % raw rice bran, T3 30 % raw rice bran, T4 20 % extruded rice bran, T5 30 % extruded rice bran

^b^Vitamin concentrations per kg of diet: vitamin A 10000 IU, vitamin D_3_ 2000 ICU, vitamin E 10 IU, vitamin K_3_ 2.5 mg, thiamin 1.5 mg, riboflavin 5 mg, niacin 40 mg, pantothenic acid 10 mg, pyridoxine 5 mg, folic acid 1 mg, vitamin B_12_ 0.01 mg, biotin 0.15 mg, choline chloride 1200 mg

^c^Mineral concentrations per kg of diet: Mn 60 mg, Zn 40 mg, Fe 80 mg, Cu 8 mg, Se 0.15 mg

^d^Based on ingredient composition in NRC (1994)

### Performance and excreta collection

Data on weekly feed intake, body weight gain, and feed conversion ratio were collected during the starter and grower periods. At 21 day of age, excreta samples, separated from feathers and scales, were collected on aluminum foil for a 24-h period, and then immediately frozen at −20 °C until future analysis. The excreta in each group were homogenized, oven-dried (60 °C for 72 h), and ground with a hammer mill (1-mm screen).

### Analytical measurements

Acid-insoluble ash as an indigestible natural marker in the diet and feces was measured using the procedure proposed by Van Keulen and Young [20]. Total P content was determined spectrophotometrically [21], and total available P was calculated as described by Scott and Balnave [22]. At the end of the experiment (42 d), the left tibia of one bird per replicate was removed and pooled for determination of tibial ash. Briefly, the tibia was separated and cleaned of meat and soft tissues; a longitudinal cut was made in tibia which was then immersed in ethyl ether for 48 h to remove excess fat. Ashing was performed at 550 °C for 7 h.

### Statistical analysis

The data were analyzed as a completely randomized design using the GLM procedure of SAS® software [23]. Mean separation was performed using the Duncan’s multiple range test at 5 % significant level. Independent comparisons were performed to compare the treatment groups.

## Results

Table 2 shows the broiler performance in terms of feed intake, body weight gain, and feed efficiency. During the starter period, feed consumption of birds receiving T1, T4 and T5 diets was significantly higher than those receiving T2 and T3 diets. However, non-significant changes in feed intake were found during the grower (22–42 d) (*p* = 0.5146) and overall (8–42) periods (*p* = 0.2484). Birds feeding on the diets containing un-extruded rice bran had a significantly lower body weight gain compared to those on the control diet (T1), T4 and T5 diets. Feed conversion ratio followed the same trend as body weight gain; birds on T1, T4 and T5 diets had a lower feed-to-gain ratio (*p* < 0.05).

**Table 2:** Effect of extruded rice bran on feed intake, weight gain, and feed conversion ratio (FCR) in broiler chicks

Performance	Diets					Statistics	
	T1	T2	T3	T4	T5	SEM	*p*-value
Feed intake (g)							
Day 8–21	633.1^a^	541.0^b^	537.3^b^	622.7^a^	605.7^a^	42.70	0.0031
Day 22–42	2483.1	2409.8	2425.8	2429.2	2418.7	107.74	0.5146
Day 8–42	3116.2	2950.8	2963.2	3052.0	3024.7	138.36	0.2484
Body weight gain (g)							
Day 8–21	504.1^a^	411.2^b^	402.0^b^	490.6^a^	477.1^a^	36.45	0.0004
Day 22–42	1102.0^a^	910.0^b^	886.2^b^	1071.5^a^	1044.5^a^	44.84	<0.0001
Day 8–42	1606.1^a^	1321.2^b^	1288.2^b^	1562.1^a^	1521.6^a^	66.72	<0.0001
FCR							
Day 8–21	1.26^b^	1.32^a^	1.34^a^	1.27^b^	1.27^b^	0.033	0.0041
Day 22–42	2.26^b^	2.65^a^	2.71^a^	2.27^b^	2.32^b^	0.090	<0.0001
Day 8–42	1.94^b^	2.24^a^	2.30^a^	1.96^b^	1.99^b^	0.076	<0.0001

*T1* control, *T2* 20 % raw rice bran, *T3* 30 % raw rice bran, *T4* 20 % extruded rice bran, *T5* 30 % extruded rice bran

^a−b^Means in the same row with different letters differ significantly (*p* < 0.05)

The effects of dietary treatments on P availability, weight and ash content of tibia, and internal organ weights are presented in Table 3. Relative to other groups, total P availability was higher in the control and T4 groups (Table 3). The P availability was 23.6 and 32 % higher in chicks fed on T4 diet compared with T2 and T3 diets, respectively. No significant difference was found in tibial weight (*p* = 0.5457). Tibial ash content did not differ significantly between T1 and T4 groups, which recorded a higher tibial ash content compared to other treatments. Extrusion pretreatment of rice bran increased the tibial ash content in such a way that it was the highest for T1 and T4 diets followed by T5 and then for those containing raw rice bran (T2 and T3).

**Table 3:** Effect of extruded rice bran on P availability, tibial weight and ash, and organ weights in broiler chicks

	Diets					Statistics	
Items	T1	T2	T3	T4	T5	SEM	*p*-value
P availability (%)	48.26^a^	37.44^c^	35.06^c^	46.29^a^	42.54^b^	2.812	<0.0001
Tibial weight^e^	0.34	0.36	0.35	0.34	0.35	0.022	0.5457
Tibial ash^f^	47.83^a^	43.81^c^	40.40^d^	47.85^a^	45.96^b^	1.312	<0.0001
Organs^e^							
Heart	0.51^c^	0.57^ab^	0.58^a^	0.50^c^	0.53^bc^	0.032	0.0034
Liver	2.20	2.29	2.34	2.25	2.21	0.111	0.2981
Pancreas	0.22^c^	0.27^b^	0.30^a^	0.23^c^	0.22^c^	0.011	<0.0001

*T1* control, *T2* 20 % raw rice bran, *T3* 30 % raw rice bran, *T4* 20 % extruded rice bran, *T5* 30 % extruded rice bran

^a−d^Means in the same row with different letters differ significantly (*p* < 0.05)

^e^Percentage of live body weight

^f^Percentage of dry bone weight

The weight of internal organs was similar in birds feeding on T1, T4 and T5 diets (Table 3). The pancreatic weight in T2 and T3 groups was higher than in other groups (*p* < 0.05). Compared to the diets containing raw rice bran, the cardiac and pancreatic weights were decreased in response to extrusion treatment (*p* < 0.05), with the birds receiving T3 diet having the greatest cardiac weight. The liver weight was not affected by treatments.

## Discussion

Published data on the broiler response to rice bran feeding are inconsistent primarily due to differences in fiber, protein and oil contents, lipid rancidity and other constituents [6, 12]. For example, Mujahid et al. [6] reported a decrease in performance and increases in organ weights of broiler chicks fed on diets containing rice bran. In another study, inclusion of 300 g/kg rice bran in broiler diets replacing corn, improved live weight gain, whereas feed conversion efficiency remained unaffected [24]. In line with our current results, Gallinger et al. [25] reported a 3.6 and 8.0 % reduction in weight gains of broiler chicks fed 30 and 40 % rice bran, respectively.

The beneficial effect of extrusion processing of rice bran on the performance of broiler chickens was reported by Mujahid et al. (2004) [6]; among the different processing methods evaluated [extrusion cooking, roasting or pelleting and treatment with antioxidant], extrusion processing resulted in better performances. In agreement with our findings, Sayre et al. (1988) reported that extrusion cooking of rice bran improved the productive performance of broiler chicks in terms of weight gain and feed-to-gain ratio in the first two weeks of feeding, which disappeared as the feeding period approached its end [18].

The tibial weight was decreased with the increasing level of rice bran in broiler diet [26]. The phytate form of P in rice bran was estimated at about 90 % of the total P with a very low availability; this forms complexes with proteins and starch, which limits calcium, magnesium, iron, and zinc availability [25, 27, 28]. According to Belyea et al. [29], availability of P in rice bran for young turkey poults was only 9 %. This limited bioavailability is primarily due to the fact that poultry lack significant amounts of endogenous phytase, the enzyme which is needed for breakdown of phytate-bound P and subsequent release of P for absorption [30, 31]. Therefore, with regard to the improved utilization of P in the extrusion-treated rice bran, it might be suggested that extrusion treatment reduced the phytin level, which subsequently makes P more available to chicks.

Greater P availability may disturb calcium to P ratio, and thus compromise the broiler performance. Therefore, an improvement in dietary calcium to P ratio with the addition of calcium to the diet containing extruded rice bran is expected to optimize the growth rate in the period beyond two weeks [18].

Mujahid et al. [6] reported that extrusion treatment of rice bran led to a decrease in the pancreatic weight of broiler chicks, but as the level of inclusion increased, the weight of pancreas also increased [6]. Contrary to our findings, Sayre et al. [18] reported no change in the weight of pancreas by extrusion cooking of rice bran. However, the pancreatic weight of birds fed on the control diet (without rice bran) was significantly lower than in birds fed on either raw or extruded rice bran diets. Trypsin inhibitors cause an excessive stimulation of the pancreatic activity and an increase in size and numbers of pancreatic cells [32]. The lower relative weight of pancreas in diets containing extruded rice bran might probably be due to destruction of trypsin inhibitors as a result of extrusion treatment, thus demonstrating the effectiveness of extrusion treatment in denaturing the trypsin inhibitor.

## Conclusions

Results of the present study confirmed the potential of extrusion treatment as an efficient processing technology in increasing the quality of rice bran for poultry nutrition. Extruded rice bran up to 30 % of the diet improved the broiler performance and P availability in broiler chicks.
